# Comparative Genetic Analysis of the Promoters of the ATG16L1 and ATG5 Genes Associated with Sporadic Parkinson’s Disease

**DOI:** 10.3390/genes14122171

**Published:** 2023-12-02

**Authors:** Ana Gómez-Martín, José M. Fuentes, Joaquín Jordán, María F. Galindo, José Luis Fernández-García

**Affiliations:** 1 Nursing Department, Faculty of Nursing and Occupational Therapy, University of Extremadura, Avda de la Universidad s/n, 10003 Cáceres, Spain; 2 Instituto de Investigación Biosanitaria de Extremadura (INUBE), 10003 Cáceres, Spain; jfuentes@unex.es; 3 Departamento de Bioquímica y Biología Molecular y Genética, Facultad de Enfermería y Terapia Ocupa-cional, Universidad de Extremadura, 10003 Cáceres, Spain; 4 Centro de Investigación Biomédica en Red en Enfermedades Neurodegenerativa, Instituto de Salus Carlos III (CIBER-CIBERNED-ISCIII), 28029 Madrid, Spain; 5 Pharmacology, Medical Sciences Department, Albacete School of Medicine, University of Castilla-La Mancha, 02008 Albacete, Spain; joaquin.jordan@uclm.es; 6 Pharmaceutical Technologic, Medical Sciences Department, Albacete School of Pharmacy, University of Castilla-La Mancha, 02008 Albacete, Spain; maria.galindo@uclm.es; 7 Animal Production and Food Science Department, Faculty of Veterinary Sciences, University of Extremadura, Avda. de la Universidad, s/n, 10003 Caceres, Spain

**Keywords:** ATG16L1, autophagy, Parkinson’s disease, polymorphism, promoter, genetic variants, sequencing

## Abstract

**Highlights:**

What are the main findings?

- Gene sequencing studies may reveal how the promoter region of the ATG16L1 and ATG5 genes is associated with sporadic PD patients.
- Most PD-related haplotypes were found for ATG16L1, suggesting a specific link between genetic variation for this promoter and the population analysed with respect to sporadic PD.

What is the implication of the main finding?

- Some mutations within haplotypes may provide valuable information on multifactorial PD.
- Consequently, the architecture of certain haplotypes and ethnicities could explain the risk of PD and the neurodegenerative process.

**Abstract:**

Sporadic Parkinson’s disease, characterised by a decline in dopamine, usually manifests in people over 65 years of age. Although 10% of cases have a genetic (familial) basis, most PD is sporadic. Genome sequencing studies have associated several genetic variants with sporadic PD. Our aim was to analyse the promoter region of the ATG16L1 and ATG5 genes in sporadic PD patients and ethnically matched controls. Genotypes were obtained by using the Sanger method with primers designed by us. The number of haplotypes was estimated with DnaSP software, phylogeny was reconstructed in Network, and genetic divergence was explored with *Fst.* Seven and two haplotypes were obtained for ATG16L1 and ATG5, respectively. However, only ATG16L1 showed a significant contribution to PD and a significant excess of accumulated mutations that could influence sporadic PD disease. Of a total of seven haplotypes found, only four were unique to patients sharing the T allele (rs77820970). Recent studies using MAPT genes support the notion that the architecture of haplotypes is worthy of being considered genetically risky, as shown in our study, confirming that large-scale assessment in different populations could be relevant to understanding the role of population-specific heterogeneity. Finally, our data suggest that the architecture of certain haplotypes and ethnicity determine the risk of PD, linking haplotype variation and neurodegenerative processes.

## 1. Introduction

Parkinson’s disease (PD) has been described as a progressive neurodegenerative disease affecting neurons in the substantia nigra (SN) involved in dopamine synthesis [1]. People over the age of 65 sometimes develop the sporadic form of the disease. Resting tremor, postural instability, rigidity, and bradykinesia [2] are hallmarks, as are nonmotor manifestations such as apathy, cognitive impairment, or dysautonomia symptoms, among others [1], although sporadic PD is attributed to genetic and environmental factors, including its interactions. Furthermore, although the specific underlying molecular pathways are largely unknown, the formation of Lewy bodies and the loss of dopaminergic neuronal cells in the SN are major pathogenic features.

Familial/genetic PD is known to constitute ~10% of cases, while sporadic PD constitutes ~90% [3]. Recent genome-wide association studies, next-generation sequencing, and exome sequencing have associated several genetic variants with sporadic PD, including leucine-rich repeat kinase 2 (LRRK2), the lysosomal β-glucocerebrosidase (GBA) gene, the microinsertion in the promoter of the ATG16L1 gene, and promoters plus SNP variations within introns in the ATG5 gene [3,4,5,6].

Autophagy is a highly conserved cellular pathway that delivers long-lived proteins and organelles to lysosomes for digestion. It consists of three events: macroautophagy, microautophagy, and chaperone-mediated autophagy [7,8]. Several animal and human studies have confirmed that dysfunctional macroautophagy and chaperone-mediated autophagy contribute to PD pathogenesis [7,9,10] through the activity of multiple autophagy proteins (ATG), with up to 31 genes being identified [11]. These genes play an important role in the different stages of autophagy, whereby abnormal expression of any gene can induce a range of pathological changes [6]. In macroautophagy (also autophagy), the key ATG proteins that can be found in this process are the following: the ATG1/ULK complex, the ATG9 cycling system, the PtdIns 3-kinase complex, the ATG12 conjugation system, and the ATG8/LC3 conjugation system [12]. ATG16L1, together with ATG5, ATG7, ATG10, and ATG12, is a major component of the ATG12 conjugation system and aids in the elongation of the phagophore, which is the precursor of the autophagosome [12,13]. In addition, ATG16L1 forms an oligomeric complex with ATG12-ATG5 conjugates to enhance LC3/ATG8 conjugation with phosphatidylethanolamine by recruiting an LC3-ATG3 intermediate [13,14] and specifies the site of LC3 lipidation [15]. Furthermore, autophagic disruption and enhanced production of IL-1β and IL-18 implicated ATG16L1 in the inflammatory immune response [15]. In addition, a polymorphic mutation of the ATG16L1 gene has been linked to retention of early-stage cells in various tissues during development as well as impaired differentiation into neurons [16]. ATG16L1 also performs no autophagic functions during cellular secretion and exocytosis [17,18].

Different levels of regulation of autophagy pre-transcriptionally, transcriptionally, and post-translationally have been demonstrated [19]. Moreover, mutations in ATG genes have been linked to various human diseases. It has been speculated that genetic variants in ATG genes may impair autophagic function, contributing to the sporadic onset of PD [20,21,22]. Previous studies have found and functionally examined some genetic variants within the regulatory domains of autophagy genes, such as microtubule-associated protein 1 light chain 3 beta (LC3B), ATG5, and ATG7, in sporadic PD patients [20,23]. In this study, we genetically analysed the crucial core promotor regions of the autophagy genes ATG16L1 and ATG5 in groups of sporadic PD patients and ethnically matched controls. In addition, we assessed the effect that molecular variation in the promoter might have on transcription using bioinformatic screening approaches aimed at studying the altered functionality of variants detected in the promoter regions of these ATG genes.

## 2. Materials and Methods

### 2.1. Study Subjects

From a total of 84 individuals, 56 patients with sporadic PD (36% females; the mean age at debut was 62.53 years and the mean sampling age was 73.30 years old, but the overall mean age of the sampling age for this study was 68.8 years) were recruited with biological material from the Servicio de Neurología del Complejo Universitario Hospitalario de Albacete del Servicio de Salud de Castilla la Mancha (SESCAM, Albacete, Spain). All PD patients were diagnosed by two neurologists. Ethnically matched healthy controls (n = 28; mean age 64.3 years and 39.1% females) were recruited from the same hospital and diagnosed as healthy. Initially, PD patients and controls with a family history of PD were excluded. This study was approved by the Human Ethics Committee of the Affiliated University of Extremadura. Informed consent was obtained.

### 2.2. Genotyping by Direct DNA Sequencing

Whole blood was used for genomic DNA isolation. DNA was extracted with Archive Pure DNA Blood Kit (5PRIME GmbH). The promoters of the ATG16L1 and ATG5 genes, from −1095 bp (Location 233250476) to +153 bp (Location 233251723) (size = 1248 bp) and from −929 bp (Location 10677904) to +90 bp (Location 106775925), respectively, were amplified using PCR and directly sequenced in both directions using the primers listed in Table 1. PCR primers were designed using the genomic reference sequence of human ATG16L1 gene promoters (GenBank Acc. No.: NC_000002.12 from 233250476-233251722 nucleotides in *Homo sapiens* chromosome 2, GRCh38.p13) and ATG5 (NC_000006.11 from 106773593-106774764 nucleotides in *H. sapiens* chromosome 6 GRCh37.p13). DNA fragments were sequenced with the Big Dye^®^ 3.1 cycling sequencing Kit (Thermo Fisher Scientific, Waltham, MA, USA), cleaned with Performa^®^DTR (Dye Removal), run on a 3130 DNA Analyzer (Applied Biosystems, Foster City, CA, USA), and compared with the wild-type ATG16L1 and ATG5 genomic reference gene promoter. All polymorphic sites were validated using BLAST searches in NCBI and compared with those observed by Wang et al. [22] and Chen et al. [20], respectively, and their rs identifiers (reference SNP cluster IDs assigned by the National Centre of Bioinformatic Institute (NCBI)) were annotated.

### 2.3. Haplotyping

The analysis of the raw data of the sequences obtained in the DNA genetic analyser (Applied Biosystems™ 3130 DNA Analyzer, Foster City, CA, USA) was carried out using the computer application “ABI Sequencing Analysis” version 5.2 (Applied Biosystems Company, Foster City, CA, USA). Ambiguous bases corresponding to mutations (SNPs) were edited, and polymorphic variations were recoded following the IUPAC nomenclature. After this, we used the DnaSP version 6.0 Bioinformatics Program [24] to estimate the minimum number of underlying haplotypes in the global data set using the “Open Unphase/Genotype data File” option following the authors’ recommendations [24]. This allowed the haplotypes of each individual to be reconstructed. This haplotype reconstruction was carried out using the PHASE, fast-PHASE, and HAPAR algorithms in the corresponding option program. Of these, the algorithm in PHASE, which uses a Bayesian fusion-based method to infer haplotypes, produced the best results using hybrid modelling (recombination and nonrecombination) [24]. Once haplotypes were reconstructed, a linkage analysis was performed by comparing all possible *loci* pairwise (e.g., n = 6 pairs for ATG16L1). Since only two alternative haplotypes were found for ATG5, it did not make sense to perform linkage analysis. In addition, the phylogeny was reconstructed to represent evolutionary events more explicitly than two-dimensional phylogenetic trees using NETWORK software version 5.0.1.1 (Fluxus-Technology Ltd., Colchester, UK).

### 2.4. Statistical Analysis

Hardy–Weinberg (EHW) equilibrium analysis was performed on each SNP and globally for both the total sample population and each group separately (patients and controls) in the GENPOP program [25] using Fisher’s exact test. *p*-values were considered significant at *p* < 0.05.

In the “Linkage Disequilibrium” command of DnaSP [24], the degree of linkage disequilibrium (LD) that can occur due to the nonrandom association (recombination frequency <50%) of nucleotide variants between different polymorphic sites located linearly in the same sequence was calculated using ATG16L1 haplotypes. The paired analysis was performed for all pairs of polymorphic sites observed in the data. The degree of LD calculated was the default indicated by the program: D [26], D′ [27], R, and R^2^ [28]. The statistical significance of LD was analysed with both the two-tailed Fisher’s exact test and the Chi^2^ test to determine whether the associations between pairs of polymorphic sites were significant [29] (*, *p* < 0.05; **, *p* < 0.01; ***, *p* < 0.001). In addition, the program checked significance for multiple testing in our case by means of the Bonferroni correction [30].

Haplotype-based genetic divergence relationships between the two groups were explored between both populations using statistics conventional pairwise *Fst* and transformed pairwise *Fst* statistics (using the matrix of proportion of differences between haplotypes as the mean number of differences per pairs of Nei within and between pairs of populations) in the ARLEQUIN program [31] to account not only for haplotype frequencies but also for molecular differences between haplotypes of ATG16L1 promoters.

In ATG16L1, we also explored whether there were differences in the mean number of mutations accumulated by each genotype because there was evidence of genetic differentiation between the genotype groups of healthy and PD patients. Two analysis designs were prepared in parallel with the aim of studying this difference. In the first design, the reference haplotype (hap1) was considered to contain zero mutations with respect to the rest because it is the wild type [22]. In a second design, hap2 was considered to contain zero mutations compared to the rest, as it is the most frequent haplotype in the whole population. The values resulting from each of the designs for the accumulative number of mutations in which each particular genotype differs were obtained as follows.

If we assigned the value 0 to the reference haplotype, then hap2, hap3, hap4, hap5, hap6, and hap7 differed from the reference by 1, 2, 2, 3, 1, and 2 mutations, respectively. If we assigned the value 0 (zero mutations) to the most frequent haplotype in the healthy population (hap2), then the haplotypes hap1, hap3, hap4, hap5, hap6, and hap7 differed from hap2 by 1, 1, 1, 2, 2, and 3 mutations, respectively. For both designs, each genotype was characterised by summing the mutation count of its two haplotypes.

In both cases, Student’s *t*-test was applied for comparison in SPSS. Groups were established using the annotations of the number of individual relative mutations from the group of PD patients and the control group. Levene’s test was applied to check the homogeneity of variances. This test indicated that the variances were significantly unequal. Consequently, the results of the Student’s *t*-test were carried out assuming unequal variances. Significance was set as *p*-values < 0.05.

### 2.5. Analysis of Transcription Factor (TF) Binding Sites in EP Promoters

Analysis of transcription factor (TF) binding sites associated with polymorphisms in PD promoters was performed using the JASPAR program [32]; thereafter, searches were refined in ConSite [33] using the inferred haplotypes.

## 3. Results

In this study, four and two single-nucleotide polymorphisms (SNPs) were observed using multilocus sequence typing (MLTS) in patients and controls with ATG16L1 and ATG5 gene promoter data, respectively, except one ins/del thoroughly studied in Gomez-Martín et al. [5]. These SNPs reached only 62.5% and 28.6% of the total found (n = 8 SNPs) in Wang et al. [22] and Chen et al. [20] for the complete ATG16L1 and ATG5 gene promoters, respectively. The SNP location and genotypic frequencies for both promoters are shown in Table S1a,b. On the one hand, the genotypic distribution for both ATG5 polymorphic *loci* in the PD and control groups did not disagree with the Hardy–Weinberg equilibrium (HWE) (Table S1a). Subsequently, we analysed whether there were differences in allele frequencies between the control and PD groups. No significant Chi^2^ test was obtained (Chi^2^ = 0.0955; *p* = 0.757), suggesting no allele differences between groups for the ATG5 promoter. These results indicated that the development of sporadic PD does not appear to be associated with the genetic variation observed in the ATG5 promoter. For this reason, it was not worthwhile to perform further analysis with this gene for this population, although it did not rule out that other mutations upstream or downstream of the sequence region analysed may be relevant. However, the two sites with polymorphic SNPs for the ATG5 promoter were studied using a BLAST search to validate all alternative alleles, and rs identifiers were assessed (see Table 2).

On the other hand, the genotypic distribution for the four ATG16L1 polymorphic *loci* in the PD and control groups did not disagree with the HWE (Table S1b), but further analysis was needed, as will be explained later. The four sites with polymorphic SNPs for ATG16L1 were studied using BLAST search to validate all alternative alleles. Moreover, the rs identifiers were assessed using Esembl version NSG00000085978.22 of the complete human genome. Since the sequences were obtained in this study, all rs identifiers that were previously recorded according to the NCBI reference database were revisited one by one in the NCBI database to validate and annotate the reference allele variant found there for each SNP (see Table 2).

DNA sequences were submitted to GenBank (Acc. Nos. ON230169 to ON230233 and OR236259 to OR236261). One novel heterozygous genotype was identified in one PD patient, but it carried an allele with a short insertion in the ATG16L1 promoter [5]. The SNPs rs1816753 and rs12476635 were found in both PD patients and controls with similar frequencies (*p* > 0.05). However, polymorphisms at rs74599577 and rs77820970 were observed only in patients but with different frequencies for the mutated T allele (Table 2), much less frequent than in the former.

**Figure 1:**
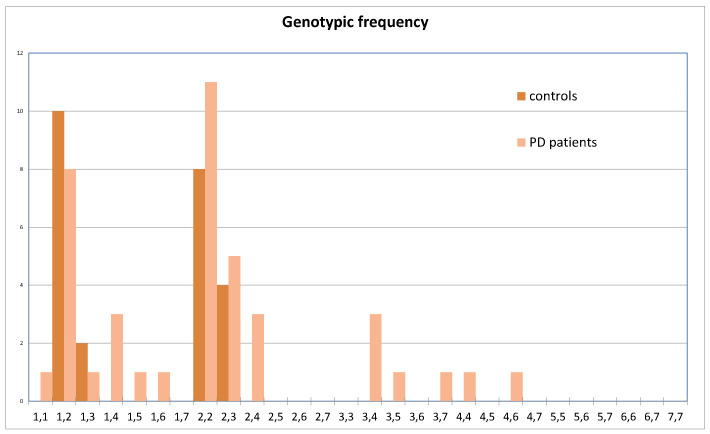
Genotypic frequency distribution using haplotypes of the ATG16L1 promoter gene. The *X*-axis indicates all possible genotypes after matching the seven (numbered from 1 to 7) haplotypes found. The *Y*-axis shows the absolute frequencies of such genotypes.

**Figure 2:**
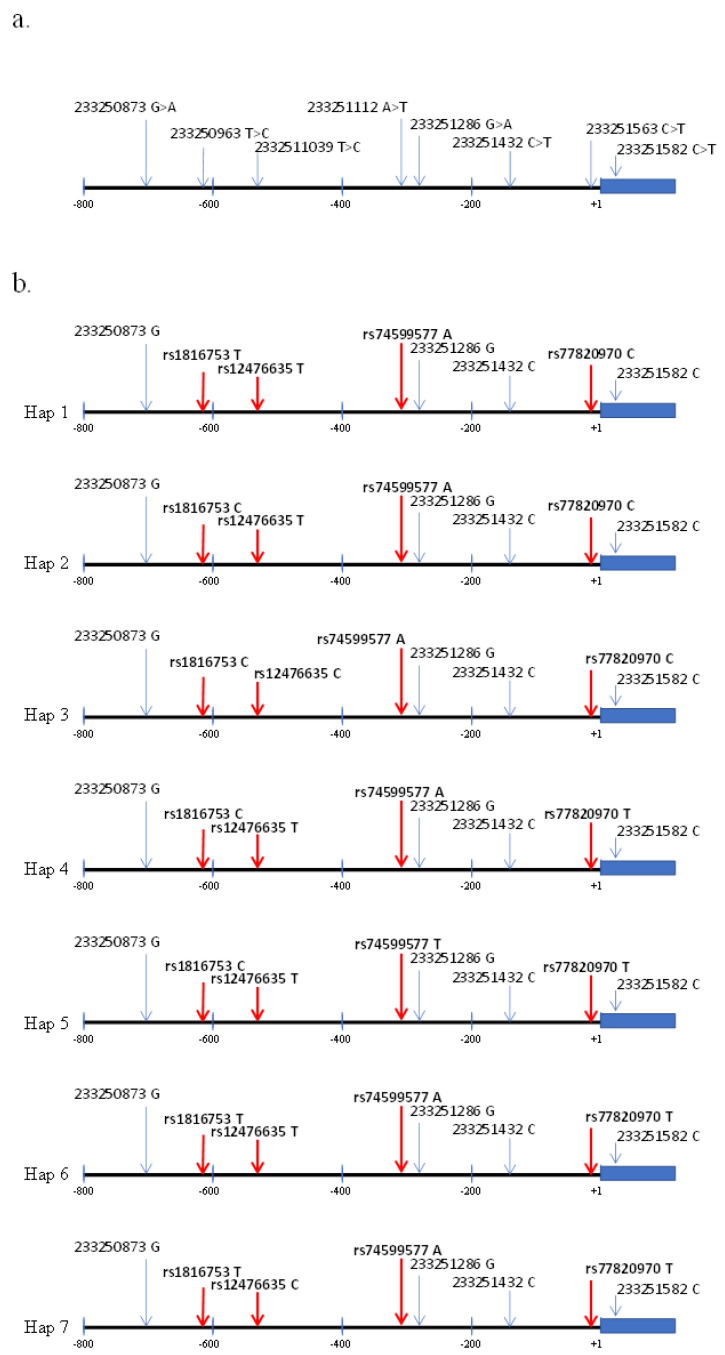
(**a**) SNPs identified in controls and PD patients according to Wang et al. [22]. (**b**) Schematic of all seven haplotypes showing the sequence variants in the promoter of the ATG16L1 gene identified in this study. The red arrows indicate the SNPs found in this study within each haplotype.

**Figure 3:**
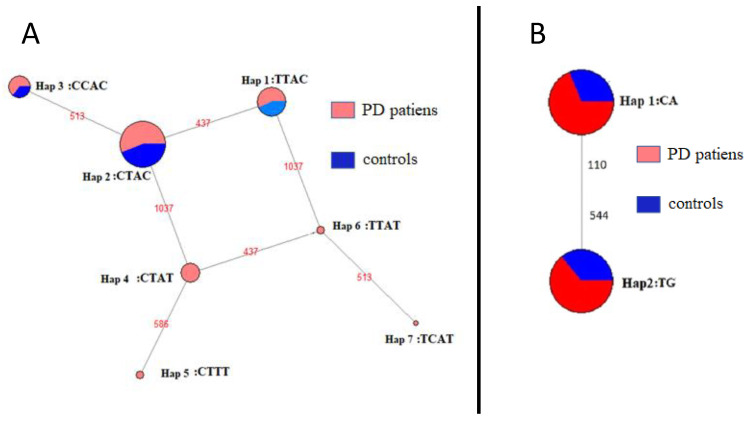
NETWORK cladogram representing the evolutionary relationship between (**A**) ATG16L1 and (**B**) ATG5 haplotypes. Each haplotype is represented by all its polymorphic sites from rs1816753 (first left C/T) to rs77820970 (last right C/T) (see Table S1 for details).

Because the sequences consisted of genotype data with four variable SNPs, a reconstruction of the haplotypes subjacent to the global data set was performed. Figure 1 shows the distribution of genotypes for ATG16L1 using these haplotypes. Seven and two haplotypes were detected for ATG16L1 (Figure 2a,b) and ATG5 (not shown), respectively. Specifically, for ATG5, strong gene linkage was observed for both polymorphic *loci* with TG (reference alleles) and CA haplotypes without recombination, even when detected yet (Table 2 and Figure 3b). Conversely, using ATG16L1 haplotypes, the linkage disequilibrium (LD) between all pairs of variable sites was estimated. Fisher’s exact test revealed no significant LD after Bonferroni correction. Allelic and genotypic differentiation between control and PD using haplotypes (see diagrams in Figure 2) was tested *locus* by *locus* using both genotypes and alleles. Significant differences were observed only for haplotypes carrying T at rs77820970 (*locus* 4: hap 4, hap 5, hap 6, and hap 7), which were only present in genotypes of PD patients (Figure 3a) at *locus* 4 (*p*-values = 0.00012 ± 0.00005 and 0.00028 ± 0.0016 for allelic and genotypic differentiation, respectively). The haplotype-based distribution of genotypes showed an absence of these haplotypes in the controls (Figure 1). Moreover, *Fst* may be a valid way to measure genetic variation, and it may be able to estimate *locus* and population-specific effects to identify genomic regions or populations with unusual evolutionary histories. Accordingly, disease-associated haplotypes between control and PD patients were further analysed using pairwise conventional *Fst* (based only on haplotype frequencies) and *Fst* (based on Nei’s average number of pairwise differences). There was a significant differentiation mainly for comparisons using the *Fst* based on Nei’s average number of pairwise differences (*p*-value = 0.02604 ± 0.0015), suggesting a higher relevance of haplotypes carrying disease-associated SNPs (especially rs77820970) in the sample population used for this study.

Phylogenetic relationships between haplotypes and identification of recombination or retromutation (hot spot) events were reconstructed by means of a median-joining network using haplotypes in the Network program (see Materials and Methods). As a result, a recombination event involving haplotypes hap1 (T-437-C-1037), hap2 (C-437-C-1037), hap4 (C-437-T-1037), and hap6 (T-437-T-1037) was detected among the most distant SNPs from the ATGL16 promoter (Figure 3).

In our NetWork analysis, each haplotype was represented by nodes connected by one single-mutation character from the nearest ones (Figure 3). All these nodes were plotted on the tree proportionally to the allele frequency of each node using the total data set. In addition, to describe the haplotypes in the nodes belonging to the different population groups of control or PD patients, they were assigned colour codes proportional to their allele frequency (Figure 3). According to our results, only haplotypes hap4, hap5, hap6, and hap7 were found exclusively in patients diagnosed with PD, sharing a T in SNP rs77820970. The remaining haplotypes were present in the controls and patients but appeared in their nodes with similar proportions.

**Figure 4:**
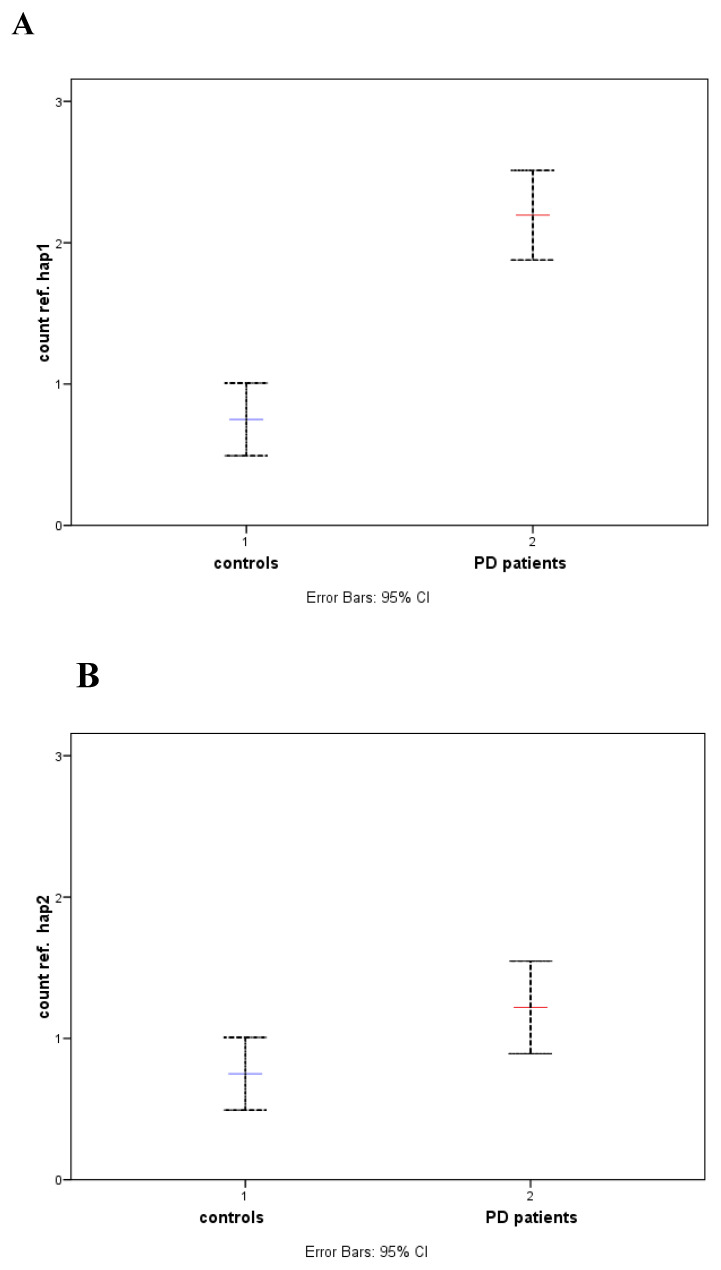
Comparison of averaged cumulative counts in controls (1) versus patients (2) against reference genotypes carrying homozygous hap1 (**A**) or homozygous hap2 (**B**). The error bar corresponds to the 95% confidence interval. Blue and red lines for the mean in controls and patients, respectively.

To further support these results, the cumulative count number of mutations attributable to each individual genotype within the groups was averaged against one of two reference genotypes (see Material and Methods). To this end, two differences in the mean number of observed mutations (Control vs. PD) were assessed by Student’s *t*-test: first, with respect to the homozygous hap1 genotype (22), and second, with respect to the homozygous hap2 genotype (the most frequent sequence in this study) (Figure 4A,B). According to the analysis, significant differences in the cumulative average number of mutations were observed between groups regardless of the haplotype used as a reference (hap1 or hap2). With either of the two haplotypes used as a reference, a lower cumulative average was obtained in patients compared to healthy patients (controls) (0.75 vs. 2.2 t_student_ = 4.339; *p* = 0.001 and 0.75 vs. 1.2 t_student_ = 5.758; *p* = 0.025 for the hap1 or hap2 reference genotypes, respectively), the difference being significant in both cases. All this suggests that there was a significant excess of mutations in the ATG16L1 gene promoter in patients that could influence sporadic PD disease in the study population, which was also supported by the results of genetic divergence and differentiation using *Fst*.

In silico analysis of the promoter region of the ATG16L1 gene with the transcription element search system using JASPAR [32] and ConSite [33] suggested changes in the transcription factor strength of binding sites, especially for AP2alpha (adapter protein 2 (AP-2)). Table 3 summarises the study of binding sites and strength for AP2alpha (adapter protein 2 (AP-2)), as it is a protein that activates the transcription of some genes and inhibits the transcription of others. This TF was obtained in all analyses, as it is involved in differences in binding forces between different haplotypes. However, although no disruptions of the binding sites were observed, the score for Loc 4 showed the greatest difference in score between hap1, hap2, and hap3 compared to hap4, hap5, hap6, and hap7. Interestingly, all these haplotypes carried T at rs77820970.

## 4. Discussion

Damaged organelles and waste macromolecules of normal cells need to be degraded using their own lysosomes by the process of autophagy, sometimes induced by various external (e.g., hypoxia or insufficient nutrition) or internal (e.g., damage or cytoplasmic aggregation) conditions [34,35]. A wide range of neurodegenerative diseases (in particular, the susceptibility of neurons to lysosomal dysfunction) are attributed to disorders of autophagy, which manifest as central nervous system dysfunction in more than two-thirds of lysosomal storage diseases [36].

The GWAS catalog contains curated data extracted from the literature, including publication information, study cohort information (cohort size, country of recruitment, and ancestry of subjects), gene information, SNP–disease association, risk allele frequency (RAF), and the assigned trait that best represents the phenotype under investigation. Up to 561 variants and risk alleles, 71 studies, and 28 full statistical summaries can be found in PD (see https://www.ebi.ac.uk, accessed on 1 September 2023. Among them, polymorphisms within ATG genes have been found to be important for PD [37,38], but less attention has been given to the role of the complete promoter regions despite their relevance as binding sites for the different kinds of TF.

This study focused on variations in the promoter of the referred gene by examining its molecular architecture in genetically unrelated Spanish PD patients to detect associations with sporadic PD but—for the first time—using both haplotypes and single SNP site analysis of the ATG5 and ATG16L1 genes.

By analysing the role of ATG5 and ATG16L1 gene polymorphisms, an association with different human diseases has been suggested [6,22], but it simplified the analysis to the *locus* (SNP) level instead of the structure of the haplotype and the resulting combination in the genotype, which remains largely unexplored as proposed in the objectives of this study.

On the one hand, a correlation between ATG5 (mapping to the *human chromosome*) and PD susceptibility remains unclear [6]. Although a link between a genetic variant within the ATG5 gene and PD has been reported [6], it also supports the negative result for rs510432 found in this study. Furthermore, a strong correlation with allele frequency, genotype frequency, and cognitive impairment and early-onset Parkinson’s disease (EOPD; onset before 50 years old) was found in PD patients carrying rs17587319. Additionally, the expression level of ATG5 in plasma was only significantly higher for EOPD patients [6]. However, late-onset Parkinson’s disease (LOPD; onset at more than 50 years old) was not significantly different than in controls. All these findings suggest that ATG5 plays a more important role in EOPD than LOPD patients [6], as supported in this study with a higher proportion of LOPD patients (81%), although we did not analyse rs17587319 because it fell out of the ATG5 promoter region [6]. However, the ATG5 rs510432 SNP has been shown to influence other diseases of the immune system associated with childhood asthma [39,40] and epilepsy associated with overdominant action on phenotypes [41]. Furthermore, ATG16L1 rs2241880 and AGT5 rs506027 polymorphisms appear to be relevant in COVID-19 [42], prompting deeper investigations. According to our study, the haplotype architecture of the gene promoters and recombination events such as those identified in ATG16 L1 should also be taken into account.

On the other hand, several studies clearly implicated SNPs of the ATG16L1 gene in different diseases. The rs2241880 (A>G) was associated with Crohn’s disease in different populations [43], as ATG16L1 regulates the specialised Paneth cells of the epithelium of the small intestine [17]. Furthermore, three mutations (rs1816753, rs12476635, and rs2289477), the first two of which are within the gene promoter, as also found in this study, were identified in a patient with acute myocardial infarction (AMI) and coronary artery ectasia (CAE). These two mutations may promote thrombosis and inflammatory responses due to abnormal dilatation of blood vessels [44]. Additionally, other gene polymorphisms have been related to carotid atherosclerotic plaques, cancer, and susceptibility to infections [45,46,47,48]. However, both the exons and introns and the promoter region of ATG16L1 gene variations have also been considered in studies of patients with PD. According to Feng et al. [19], almost all mutations were in the noncoding part of the genome, suggesting that these mutations are likely to influence the regulation of gene expression, leading to disease development [49].

In addition, the human ATG16L1-derived protein has been extensively studied, as it is needed to regulate autophagy prerecruitment structure (PAS) and autophagy activity [50]. For example, the most recent studies consistently supported a novel role of the axis V-ATPase-ATG16L1 (through its WD40 domain) in lysosomal homeostasis via LRRK2 recruitment [51]. However, few studies have been reported regarding the functional composition of the promoter region of the gene in the context of PD. Exceptionally, an extensive study by Wang et al. [22] characterised the promoter in 151 patients from Asia [22], but little is known regarding European populations such as those studied here. In addition, less is known about its expression and regulation [22]. Seven polymorphic sites were found by Wang et al. [22], of which only four were found in this study. Our results also differed from other reports [22]. We observed all genotypic classes in rs1816753, rs12476635, rs74599577, and rs77820970, especially relevant in PD patients. In contrast, only rs1816753 and rs12476635 showed all genotypic classes in Wang et al. [22]. Furthermore, the mutant types at rs74599577 and rs77820970 were always among the rarest alleles in any of the reports ([22], this study), but the latter was exclusively and significantly associated with PD patients in our study.

For the first time, up to seven haplotypes were reconstructed using the four mutations we reported, but PD patients showed only four of them. Thus, it can be suggested not only that molecular profiles differ between ethnic groups but also that haplotype architecture can be expected to vary from one ethnic group to another with respect to sporadic PD patients. This encourages further studies using full-length promoter haplotypes. Recent studies have shown a genotype–phenotype correlation of MAPT haplotypes in Parkinson’s disease (PD) wherein certain haplotypes (such haplotype H1) are linked to particular cognitive domains, including memory and visuospatial function [47]. However, the association between haplotypes and several cognitive functions in PD remains unclear, as no specific regional degenerations or neurochemical alterations have been reported [52]. Despite this, Pascale et al. [52] highlighted that the effects of particular genotypes could be detectable even in a relatively small number of subjects, as in this study.

Although direct regulation of ATG16L1 is mediated by the vitamin D receptor and several miRNAs in human cells and cell lines [53], contactin-associated protein-like 3 (CNTNAP3), which mediates neuron–glial interactions, upregulates ATG16L1 expression [54], and protein phosphatase 1 (PP1) and casein kinase 2 (CSNK2), which regulate the phosphorylation of ATG16L1 in cardiomyocytes [47], suggest that it has an important role in several tissues. For example, it has been reported that overexpression of the ATG16L1 gene occurs in patients with squamous cell carcinoma [55]. In this study, we analysed (genetically and “in silico” functionally) the ATG16L1 gene promoter and identified some genetic variants and haplotypes that may provide insights for understanding the transcriptional relevance of the ATG16L1 gene in human development and disease.

The human autophagy system involves hundreds of proteins interacting as a network organization during the autophagy process with many ATG (ATG3, ATG5, ATG10, ATG12, and LC3) proteins interacting with ATG16L1 [56] and other proteins, such as nucleotide-binding oligomerization domain containing 2 (NOD2) [57]; the Golgi-resident small GTPase Rab33 [58]; the focal adhesion kinase (FAK) family interacting protein of 200 kDa (FIP200), which is needed for autophagosome formation [59,60]; lysosome-localised TECtonin **β**-propeller repeat containing 1 (TECPR1), which forms a mutually exclusive complex with the ATG12–ATG5 conjugate [61]; the human transmembrane protein TMEM59, which promotes local activation of LC3 [62]; eva-1 homolog A (EVA1A)/transmembrane protein 166 (TMEM166), which associate during autophagosomal membrane development [63]; members of the human WD-repeat protein interacting with the phosphoinositide (WIPI) family that directly bind to ATG16L1 to recruit the ATG12–ATG5–ATG16L1 complex during the formation of autophagosomes [64]; and the PX-BAR protein SNX18, which facilitates recruitment of ATG16L1 [65]. Although Wang et al. [22] did not identify functional ATG16L1 genetic variants or factors regulating ATG16L1 gene expression, some significant particularities have been observed with respect to *locus* 4 as follows: (1) they contain the mutated form (rs77820970 T); (2) they belong to the molecular architecture of the hap4, hap5, hap6 and hap7 haplotypes, found only in patients; and (3) this *locus* explains much of the genetic divergence between the controls and PD. Therefore, although we have not collected information on transcriptional activity using experimental methods such as those carried out by Wang et al. [22], we cannot yet rule out that some variants in the ATG16L1 promoter contribute significantly to the sporadic development of PD. In this sense, Wang et al. [22] declared that although they did not identify functional genetic variants of ATG16L1, it would be convenient to carry out additional studies in PD patients to genetically analyse the proteins that interact with ATG16L1 as well as the factors that regulate the expression of the ATG16L1 gene. Indeed, Pascale et al. [52] argued that certain haplotypes may interact synergistically with other genetic variants to influence PD risk.

It has been argued that there is a link between ethnicity and the genetic architecture of haplotypes [48], so ethnicity is worth considering in the study of the association between genetic variants and the neurodegenerative process in PD, as reported in this study. In fact, the T allele (rs77820970) was associated with PD patients at a nonnegligible frequency (22%) in this study. Furthermore, if confirmed, this result may indicate that several haplotypes carrying this T allele might increase the risk of developing sporadic PD disease, at least in populations of southern European ancestry, as has been reported by Pascale et al. [51] for some haplotypes of MAPT genes. In addition, it was also corroborated in one Asian patient [22]. Phenotypic expression of PD in different populations could be relevant to the understanding the role of population-specific heterogeneity, as confirmed by large-scale evaluation [51]. Our study supports the hypothesis that genetic variability in the ATGL16L promoter region plays a relevant role in the development and progression of human diseases. It also provides a genetic basis for future research on the molecular mechanisms and the relevance of genetic analysis in PD. In addition, PD research may require knowledge and molecular isolation of the different complete haplotypes of the promoters involved for the purpose of subsequent “in vitro” functional expression studies. This would represent a significant advance in the research and exploration needed to verify the influence of mutations in the promoters, as was partially explored by Chen et al. [20].

However, as stated by Pascale et al. [52], the main limitation of this study was the small sample size; therefore, the results should be interpreted with caution until further studies with larger series of patients shed more light on the importance of specific haplotypes in sporadic PD. However, case studies highlight the importance of reporting the association between haplotypes and sporadic PD [44].

Therefore, knowledge of both point mutations and haplotypes of PD genes not only provides a better understanding of the relationship between genes and phenotypes in PD but also facilitates the discovery of new strategies to potentially lead towards investigations to further understand the clinical relevance of genes in their full entity and the molecular basis connecting genotypes and phenotypes, and it essentially increases our understanding of PD. This will also drive the future development of genetic testing assays to identify patients at risk, a goal of precision medicine. Furthermore, the wide range of SNPs and related markers inherited as linked blocks of SNPs such as haplotypes [this study] has led to increased recognition of the potential for their application to understand the genetic basis of complex traits. Many genomic methods, which also use linkage disequilibrium (LD), are now an important source of active research and gene discovery in human medicine and health studies and predictions that are recomputed as data from more patients become available [66]. Finally, all these findings have generated a demand for training in research, employment, and genome sequencing technology.

## Figures and Tables

**Table 1:** Primers to amplify the promoters of ATG16L1 and ATG5 for conventional PCR and sequencing.

PCR Primers.	Sequence	Sequence Location	PCR	Product PCR
ATG16L1 F	5′-TTCATCTCCCCCTTTCAACAT	1 a 21	Conventional	1246 bp
ATG16L1 R	5′-GAGCTCACCTCCACACACTG	1227 a 1246	Conventional	
SeqATG16L1 F	5′-CAACATCTACAGCCTCAGATTACC	16 a 39	Sequencing	1222 bp
SeqATG16L1 R	5′-CTCCACACACTGGCAGTCC	1220 a 1238	Sequencing	
ATG5 F	5′-TTCCCAATTATCAAGAACCTGTTT	140 a 163	Conventional	1021 bp
ATG5 R	5′-TCTGGTATCCAGCGAATACAACC	1160 a 1138	Conventional	
SeqATG5 F	5′-GTTTTGAGTCTCAGCACAGTAC	160 a 181	Sequencing	774 bp
SeqATG5 R	5′-ACCCTCTTCTGAGAATCTTGC	932 a 912	Sequencing	

**Table 2:** Allele frequencies observed for each *locus* in the ATG16L1 and ATG5 promoter (this study (control, patient, and global) and NCBI reports (European/total human population)), including an NCBI allele search. (reference allele > alternative allele). Variants not found in this study are in braquets parentheses.

NCBI Search	Alleles	Control	Patient	Global	European/Total Human Population
ATG16L1 promoter					
233250963T>C [A,G] (rs1816753)	T	0.250	0.232	0.238	0.305/0.299
	C	0.750	0.768	0.762	0.695/0.700
2332511039T>C (rs12476635)	T	0.875	0.854	0.862	0.861/0.856
	C	0.125	0146	0.138	0.139/0.144
233251112A>T (rs74599577)	A	1	0.976	0.985	0.988/0.989
	T	0	0.024	0.015	0.011/0012
233251563C>T (rs77820970)	C	1	0.780	0.862	0.915/0.926
	T	0	0.220	0.138	0.085/0.074
ATG5 promoter					
106774464T>C (rs510432)	T	0.518	0.536	0.521	0.542/0.511
	C	0.482	0.464	0.485	0.458/0.489
106774030 G >A (rs506027)	G	0.518	0.536	0.521	0.529/0.472
	A	0.482	0.464	0.485	0.471/0.528

**Table 3:** Summary of score results for all variable loci of ATG16L1 according to ConSite. Multiple scores correspond to multiple locations for AP2alpha at each site.

Loc1	Loc2	Loc3	Loc4	Loc1 Score	Haplotype	Loc2 Score	Haplotype	Loc3 Score	Haplotype	Loc4 Score	Haplotype
T	T	A	C	−1.747	1,**6,7**	6.121	1,2,**4,5,6**	2.135	1,2,3,**4,6,7**	2.585	1,2,3
				−0.399		7.219					
				−0.339		5.404					
C	C	T	T	−0.788	2,3,**4,5**	7.802	3,**7**	4.104	**5**	−0.063	**4,5,6,7**
				1.125		8.383					
				2.393		6.819					

## Data Availability

On request. Genotypes were disposable at NCBI (see accession numbers in the text).

## Supplementary Materials

The following supporting information can be downloaded at: https://www.mdpi.com/article/10.3390/genes14122171/s1, Table S1: Variation in the ATG5 and ATG16L1 promoter gene sequences.
