# Supplementary material for: Comparative Genetic Analysis of the Promoters of the ATG16L1 and ATG5 Genes Associated with Sporadic Parkinson’s Disease
Source: Genes (Basel). 2023 Dec 2;14(12):2171. doi: 10.3390/genes14122171 (PMC10743014; doi:10.3390/genes14122171)
Supplement: Supplementary file 1 [file genes-14-02171-s001.zip › genes-2700592-supplementary.pdf]

Supplementary Materials: The following are available online, Table S1: Variation of the ATG5 and ATG16L1 promoter gene sequence.

Table S1a. Variation of the ATG5 promoter gene sequence.

DSVs (Chen et al. 2013)	Location		Genotype	Control (n=28)		p-value	Enfermos (n=56)		p-value	Global p-value
	(upstream)	Segment		obs	exp		obs	exp		
106774464C>T (rs510432)	-769 bp	110	TT	7	7.382	1.000	14	11.946	0.292	0.655
			TC	15	14.236		24	28.108		
			CC	6	6.381		18	15.946		
106774459T>A	-764 bp	115	TT	28	-	-	56	-	-	-
106774423C>A	-728 bp	151	CC	28	-	-	56	-	-	-
106774418C>A	-723 bp	156	CC	28	-	-	56	-	-	-
106774382C>A	-687 bp	192	CC	28	-	-	56	-	-	-
106774206G>A	-511 bp	368	GG	28	-	-	56	-	-	-
106774030A>G(rs 506027)	-335 bp	544	GG	7	7.382	1.000	14	11.946	0.292	0.655
			AG	15	14.236		24	28.108		
			AA	6	6.382		18	15.946		

Table S1b. Variation of the ATG16L1 promoter gene sequence.

DSVs (Wang et al. 2017)	Location (upstream)	Segment	Genotipo	Control (n=24) obs	Control (n=24) exp	p-valor	Enfermos. (n=41) obs	Enfermos. (n=41) exp	p-valor	Global p-valor
233250873G>A (rs146693112)	-698bp	347	GG	24			41			
233250963T>C (rs1816753)	-608bp	437	TT	0	1.404	0,272	2	2.111	1.000	0.626
			TC	12	9.191		15	14.778		
			CC	12	13.404		24	24.111		
2332511039T>C (rs12476635)	-532bp	513	TT	18	18.319	1,000	30	29.815	1.000	1.000
			TC	6	5.361		10	10.370		
			CC	0	0.319		1	0.815		
233251112A>T (rs74599577)	-319pb	586	AA	24	-		39	39.012	1.000	
			AT				2	1.975		
			TT				0	0.012		
233251286G>A (rs539735288)	-285pb	760	GG	24			41			
233251432C>T (rs276250824)	-139pb	906	CC	24			41			
233251563C>T (rs77820970)	-9pb	1037	CC	24			26	24.889	0.365	
			CT				12	14.222		
			TT				3	1.889		
233251582C>T (rs1447432348)	+12bp	1058	CC	24			41			

The results of genetic diversity in controls and patients with PD are shown. Sample sizes and locations are provided according to Chen et al (20) and Wang et al. (2017) (32) (up-stream) and the location with respect to the amplified segment in this work (segment), the observed genotypes and their frequencies both observed (obs.) And expected (esp.). The p-values of the EHW tests are shown for controls, sick and both globally. In shadow-gray the non-polymorphic positions of this work are shown. The negative sign indicates the relative position of the nucleotides that are part of the promoter, and therefore are not transcribed, with respect to the beginning of the transcription and with a positive sign those positions that are outside the promoter and, therefore, specify regions that belong to the ATG5 or ATG16L1 protein transcript.
